# Touch deprivation in female adolescents: implications for semantic processing and cognition

**DOI:** 10.3389/fpsyg.2024.1504060

**Published:** 2025-01-15

**Authors:** Amjad Sohail, Salma Naz Khattak

**Affiliations:** Department of Social and Behavioral Sciences, National University of Medical Sciences, Rawalpindi, Pakistan

**Keywords:** body, touch deprivation, semantic processing, cognition, female adolescents

## Introduction

Touch is a nonverbal means of communication at the same time it is a basic necessity of emotional connection and therefore necessary for the wellbeing and social interaction of humans (Gallace and Spence, 2010). The sense of touch is integral to the human experience, meaning it is vital and prone to multiple interpretations (Stevens et al., 2024). It is not just a choice; rather, it is an innate human need. Touch activates a complex network of nerves that support various physiological functions, such as improved sleep, immune system stimulation, and digestion (Nuszbaum et al., 2014; Kulkarni et al., 2010). The human body is naturally made to respond to touch, largely via the activation of mechanoreceptors in the skin, which transmit the signals via afferent nerves to the central nervous system (Olausson et al., 2002; Morrison et al., 2011; McGlone et al., 2016). This feeling of touch not only engages neuronal circuits but also activates them, like the insular cortex and the somatosensory cortex. Both are essential for understanding the social brain hypothesis. According to this hypothesis, these circuits are essential to understand social interaction and affiliative behaviors, as they highlight the evolutionary importance of touch in building social relationships (Dunbar, 1998; Rolls, 2010).

This study builds the foundation and explores the impact of touch on semantic processing as well as on cognitive functioning because absence of touch can lead to deprivation of touch, which affects poorly on social and emotional growth (Field, 2001; Cascio et al., 2019; Suvilehto et al., 2023). Children raised in institutions are at higher risk of developing an insecure attachment style due to the lack of contact (touch) with a primary caregiver (Davis et al., 2017). In pandemic, reduction in physical touch was associated with increased anxiety, depression, and stress, with notable declines in mood and heightened loneliness (Grandi and Bruni, 2024). So, touch is not only physiologically beneficial, psychologically it fosters a sense of safety, community engagement, and emotional support (Fields, 2011; Gallace and Spence, 2010). Moreover, understanding and use of touch among individuals are profoundly shaped by sociocultural practices, and deeply rooted religious ideologies (Khattak et al., 2024). Khattak et al. further explore that patriarchal society also limits the access of female adolescents to physical gatherings to fulfill their touch needs or express them openly hampering their semantic processing, which contributes to cognitive and emotional behaviors (Jablonski, 2021). Therefore, it is critical to assess how these limitations impact female adolescents' semantic processing and cognitive development in conservative societies.

## Touch in semantics

Semantics is an important branch of linguistics that studies how meanings are created, interpreted, and understood within language. It looks at the meanings of words, phrases, sentences, and texts (Frawley, 2013), and has historically focused more on spoken language, exploring how the context, the syntax, and the pragmatic elements impact meaning. Fields such as lexical semantics examine word relationships, while compositional semantics examines how word meanings come together to form sentences (Lyons, 1995; Saeed, 2015). Nonetheless, the notion of semantics can encompass nonverbal means of communication including touch in addition to verbal communication (Madella et al., 2023). For instance, depending on the cultural and situational context, a handshake or an embrace can convey a variety of feelings and intentions, from friendship and support to power dynamics (Hertenstein et al., 2006; Gallace and Spence, 2010). For example, the Japanese often avoid hugging as it is perceived as too intimate, whereas Latin Americans embrace warmly, and it is a common practice of greeting there. In Western culture, a handshake is a professional gesture in the workplace; yet, in India and even in the Sindh province of Pakistan, greeting with folded hands to show respect. These nonverbal forms of communication are deeply rooted in human interaction, where the meanings conveyed by touch are as context-dependent and culturally variable as those of spoken language, illustrating that touch, much like language, plays a crucial role in conveying meaning and fostering understanding in social interactions (Burgoon et al., 2021). Conversely, cultures with frequent touch may have a more literal touch-related vocabulary, reflecting the direct and frequent nature of physical contact in daily life (Guest et al., 2011).

Moreover, theories of embodied cognition have gained significant influence, with research highlighting the centrality of sensorimotor experiences in cognitive and language processing (Wellsby and Pexman, 2014). In language processing, specifically semantics processing, the meanings of words are deeply rooted in context referencing and cultural sensory-motor practices. For instance, initial referencing of the words *soft* and *rough* is tangible and gives physical sensations but their meanings are conveyed metaphorically, as a *soft voice* or a *rough day*. This progression in language illustrates how embodied experiences (including touch) influence both abstract thinking and semantic processing (Williams and Bargh, 2008). It enables every individual to interpret the language and use it in complex ways. This process of semanticizing language plays an important and expressive role between sensory experiences and higher-order cognitive functions. In the process of thinking, abstract concepts connect with physical experiences. This connection highlights the embodied cognition. Consequently, embodied cognition facilitates the integration of language, thought, and social interactions. Given its important role, there is a dire need for empirical investigations to explore the mechanisms and implications of this in lab settings.

## Cognitive functioning

In orphanages, specifically newborn babies consistently lack physical touch from caregivers (Spitz, 1945; Ainsworth, 1979; Harlow and Zimmermann, 1958). Such newborn babies experience delayed physical growth and cognitive impairments, weaker immunity, and emotional dysregulation (Fields, 2011). In the 1980s, a study was conducted in Romanian orphanages, where attachment disorders and delayed growth were observed in the children who received no or little touch and were also socially isolated (Nelson et al., 2007). This phenomenon, which is later known as *failure to thrive*, warns, that lack or no touch is a critical issue that reinforces various psychological and cognitive dysfunctions (Oster, 1978; Valentijn et al., 2005).

The experience of limited or no touch affects mental health, which further leads to depression and anxiety (Croy et al., 2016). Its effects show poor academic performance and social engagement specifically in young individuals. The development of cognitive deficiencies has far-reaching societal implications, and potentially impacts the human life cycle, i.e., from infancy to old age. A disrupted social life, linked to loneliness, can lead to negative health issues such as heart disease, strokes, anxiety, depression, and cognitive conditions like dementia. Touch helps reduce such health issues. When a body is touched, deep inside the brain, the hypothalamus produces oxytocin, a hormone related to bonding and relaxation (Cascio et al., 2019). Oxytocin plays an important role in the childbirth process. Gaining a deeper understanding of oxytocin helps individuals take better care of their health and better insights into body functions (Uvnas-Moberg, 1998).

Cognitive functions, such as attention, memory, and problem-solving are interconnected with sensory inputs (Nadeau, 2020; Pulvermüller, 2005; Diamond, 2013). Research shows that touch experience strengthens the memory retrieval and formation processes by establishing connections between physical sensations and abstract concepts (Hargreaves et al., 2015). Touch experience illustrates touch semantics that shape our language comprehension and maintain cognitive performance, specifically, in decision-making (Engelen et al., 2011).

## Impacts and insights in the context of Pakistan

In the Pakistani patriarchal society, females are restricted from participating in social activities, including healthy physical activities in institutional settings (Laar et al., 2019; Ali et al., 2011). These restrictions impact the female adolescents' perceptions and experiences of their daily life opinions to express them. Even they have no autonomy to express their physical and sexual needs with their life partners, forced by religio-cultural narratives. Moreover, there is a lack of institutional support for females. Their voices for their rights are silenced and systematically ignored under the exalted ideal of social or cultural modesty (Khattak et al., 2024). These social, cultural, or religious limitations (Montagu, 1971; Gallace and Spence, 2010), aggravate mental and physical health issues, specifically, touch semantics disruptions to understand and interpret their touch experiences. This complicates social communication and relationship dynamics, not understanding the issues or misinterpreting them damages social cognition (Henley and Patrick, 1977; Field, 2001). Resultantly, these issues hamper professional effectiveness and social integration, ultimately impacting social cohesion as well as economic productivity (Bloom and Grant, 2005).

A society that limits touch for its female population may experience cognitive dysfunction, evidenced by significant social issues such as domestic violence, child abuse, and honor killings. The most severe consequences of these limitations manifest in intellectual deficiencies, slow learning, and an unskilled workforce (Field, 2001). Additionally, cultural norms often serve as a barrier to touch experience, with Pakistani society being an example where strict norms governing physical contact are often shaped by religious narratives (Ali et al., 2011). As a result, the language developed for touch semantics in such societies differs from that of Western cultures, with a more limited vocabulary related to touch, further hindering the full recognition and expression of this fundamental human need. Such an example is also seen in other conservative Asian cultures too, where hugging is not common. Specifically, in South Asia at the workplace, people avoid patting on the back as a supportive gesture, though it boosts morale in Western workplace culture. In the Middle East, due to gender norms, healthcare interaction limits therapeutic touch impacting less emotional care. The culture of United Arab Emirates discourages public display of affection, having minimal linguistic expressive frameworks to discuss their emotions. Similar discouraging emotional values are seen in Japanese culture, where suicide is prevalent (Davies and Ikeno, 2011). So, this shortage of touch vocabulary hinders well-being's emotional value.

## The way forward

Hence, the absence of physical contact interferes with the semantics of touch, impairing the ability to interpret and respond to tactile stimuli, which in turn hinders both social communication and cognitive development (Van Stralen et al., 2011). If these issues are not addressed in conservative societies, the aspiration to build a society grounded in critical thinking and social awareness will remain out of reach, exacerbating social inequalities and potentially contributing to an increase in incidents of harassment (Khattak et al., 2024). The need to confront this matter is important. Therefore, government entities, policymakers, academic institutions, and health organizations must pay attention to this concern. It is crucial to address the need for touch and emotional connectivity to cultivate a more unified and supportive society. Neglecting these essential needs could perpetuate cycles of disadvantage and imbalance, undermining societal welfare and stability (Maslow, 1943).

Educational institutions may address touch deprivation through a range of interventions. Incorporating structured physical activities such as sports, dance, and theater into the curriculum will provide students with essential tactile experiences, which are crucial for mitigating the adverse effects of touch deprivation (Palacios, 2024). Sports like soccer, basketball, or cricket and group dances (swing, ballet, cha-cha-cha, rumba, salsa, etc.), specifically folk dances encourage teamwork and physical interaction. Community schools in Brazil introduced samba into its curriculum (Raphael, 1990), and in the UK theater-based intervention helped children with autism spectrum disorder (ASD) (Beadle-Brown et al., 2018), improve their cognitive communication skills. Yoga is introduced in Indian schools, resulting in enhanced focus among students, and also decreased anxiety (Verma et al., 2014). By embedding these activities into the school environment, educational institutions can counteract touch deprivation, promote healthy development, and create a supportive, interactive learning atmosphere. The sensory stimulation derived from these activities can enhance touch semantics, enriching students' understanding and use of tactile language in everyday interactions. In this regard, educators and trainers need to be culturally aware when addressing touch deprivation. In multicultural settings, educational programs should include training on the diverse cultural norms related to touch and develop strategies that are inclusive of various cultural practices. This helps ensure that interventions and support systems are culturally sensitive and effective in meeting the needs of individuals from different backgrounds (Gallace and Spence, 2010; Anderson and Fenichel, 1989: Lopez et al., 2024; Lee and Choi, 2023).

Finally, involving parents and the broader community in supporting tactile experiences can enhance the effectiveness of these interventions beyond the school setting. Offering workshops and informational sessions for parents on how to create sensory experiences at home not only with their female adolescents but in general with their children can help address touch deprivations, especially in patriarchal societies. Additionally, establishing community partnerships to provide supplementary resources can further support these efforts. Such initiatives are essential for improving adolescents' cognitive and emotional wellbeing and ensuring that the benefits of tactile experiences are extended into their daily lives (Salthouse, 2019).

## Conclusion

Deprivation of touch is not merely a minor disturbance or distraction of an individual's routine life; rather it can create a ripple of consequences that can impact individual's future goals, relationships, and overall life trajectory. In eastern society, it is significantly impacting female adolescents by limiting their ability of free decision-making of their life matters. This deprivation leaves them cognitively and emotionally vulnerable resulting in low self-esteem, which in turn hinders them from asserting their boundaries and life choices. Insufficient touch experiences reduce their confidence to express intimate needs and weakens their resilience toward societal pressures like forced marriages, domestic violence, or denial of educational rights. To break the cycle of touch deprivation, now it is essential to empower young females through culturally appropriate interventions, thereby unleashing their full potential and fostering a bright future.
